# Retraction statement: Wu, H., He, Y., Chen, H., Liu, Y., Wei, B., Chen, G., Lin, H. and Lin, H. (2019), LncRNA THOR increases osteosarcoma cell stemness and migration by enhancing SOX9 mRNA stability. FEBS Open Bio, 9: 781–790.

**DOI:** 10.1002/2211-5463.13621

**Published:** 2023-05-23

## Abstract

https://doi.org/10.1002/2211-5463.12620

The above article, published online on 2 March 2019 in Wiley Online Library (wileyonlinelibrary.com), has been retracted by agreement between the Editor‐in‐Chief, FEBS Press, and John Wiley and Sons Ltd. The retraction has been agreed following an investigation into concerns raised by a third party, which revealed inappropriate duplications between this and another article [1]. Thus, the editors consider the conclusions of this manuscript substantially compromised.

Reference

1 Shao L, Zhang X and Yao Q (2020) The F‐box protein FBXO11 restrains hepatocellular carcinoma stemness via promotion of ubiquitin‐mediated degradation of Snail. *FEBS Open Bio* **10**, 1810–1820.doi: 10.1002/2211‐5463.12933

https://doi.org/10.1002/2211-5463.12620

The above article, published online on 2 March 2019 in Wiley Online Library (wileyonlinelibrary.com), has been retracted by agreement between the Editor‐in‐Chief, FEBS Press, and John Wiley and Sons Ltd. The retraction has been agreed following an investigation into concerns raised by a third party, which revealed inappropriate duplications between this and another article [1]. Thus, the editors consider the conclusions of this manuscript substantially compromised.

**Reference**

1 Shao L, Zhang X and Yao Q (2020) The F‐box protein FBXO11 restrains hepatocellular carcinoma stemness via promotion of ubiquitin‐mediated degradation of Snail. *FEBS Open Bio* **10**, 1810–1820.doi: 10.1002/2211‐5463.12933

